# MiR-571 affects the development and progression of liver fibrosis by regulating the Notch3 pathway

**DOI:** 10.1038/s41598-021-00638-3

**Published:** 2021-11-08

**Authors:** Shuo Cong, Yongmei Liu, Yi Li, Yu Chen, Rui Chen, Baofang Zhang, Lei Yu, Yaxin Hu, Xueke Zhao, Mao Mu, Mingliang Cheng, Zhi Huang

**Affiliations:** 1 School of Basic Medicine Sciences, Guizhou Medical University, 9 Beijing Road, Guiyang, Guizhou China; 2 Clinical Laboratory Center, Guizhou Cancer Hospital, 1, Beijing West Road, Guiyang, Guizhou China; 3 Clinical Laboratory Center, The Affiliated Hospital of Guizhou Medical University, 28, Guiyi Street, Guiyang, Guizhou China; 4 College of Medical Laboratory, Guizhou Medical University, 28, Guiyi Street, Guiyang, Guizhou China; 5 Department of Acupuncture and Moxibustion, The Affiliated Hospital of Guizhou Medical University, 28, Guiyi Street, Guiyang, Guizhou China; 6 Department of Infectious Diseases, The Affiliated Hospital of Guizhou Medical University, 28, Guiyi Street, Guiyang, Guizhou China; 7 Department of Obstetrics and Gynecology, Maternal and Child Health Hospital of Guiyang Province, 63 Ruijin South Road, Yunyan District, Guiyang City, Guizhou Province China; 8 Prenatal Diagnosis Center, The Affiliated Hospital of Guizhou Medical University, 9 Beijing Road, Guiyang City, Guizhou China; 9 Department of interventional radiology, the Affiliated Baiyun Hospital of Guizhou Medical University, Guiyang, 550005 P. R. China

**Keywords:** Cell biology, Molecular biology

## Abstract

Exploring the expression of miR-571 in patients with liver fibrosis and its role in the progression of liver fibrosis. A total of 74 patients with liver fibrosis in our institution from September to December 2018 were collected for study, and the expression of miR-571, Notch3 and Jagged1 in patients with different progressions of liver fibrosis was determined by RT-PCR and Western blot analysis. Set up Notch3 up group and Notch3 down regulated group, RT-PCR and Western blot were used to determine the effect of Notch signaling on the expression of fibrogenic factors. CCK-8, cell scratch assays, Transwell assays, flow cytometry were used to determine the effect of miR-571 on LX-2 proliferation, migration, apoptosis in human stem stellate cells, and RT-PCR, Western blot assays were performed to determine the effect of miR-571 on the Notch3 signaling pathway and the expression of profibrogenic factors. miR-571, Notch3 and Jagged1 are up-regulated in patients with liver fibrosis and is associated with the progression of liver fibrosis. Notch3 signaling pathway can promote the expression of fibroblast in human hepatic stellate cells; miR-571 can inhibit the apoptosis of human hepatic stellate cells, promote cell proliferation and migration; up regulation of miR-571 can promote the expression of Notch3 and Jagged1, and up-regulation of miR-571 also promoted the expression of related fibroblasts. MiR-571 can promote the activation of human stem cell stellate cells and the expression of fibroblast related factors through Notch3 signaling pathway.

## Introduction

Liver fibrosis is not an independent disease, but a liver disease associated with a variety of chronic liver diseases. In recent years, the incidence rate of liver fibrosis has been increasing in China. If not treated in time, there will be a large number of fibrous tissue hyperplasia in the liver, which will further develop from liver fibrosis to cirrhosis, and eventually seriously threaten the life and health of patients^1^. Hepatic stellate cells (HSCs) are located between hepatic parenchymal cells and sinusoid endodermal cells, and are normally in a static state with irregular characters. Normally, HSC is involved in vitamin A metabolism, fat storage, and the construction of extracellular matrix in the liver. HSC is activated by hepatocytes, Kupffer cells, endothelial cells, infiltrating inflammatory cells, etc., when the liver parenchyma is chronically injured. The activation of HSCs is the central link in the formation of liver fibrosis^2^.

MicroRNAs (microRNAs, miRNAs) are a class of endogenous, noncoding small molecular RNAs with regulatory functions. The abnormal expression of miRNA can affect the differentiation, proliferation and apoptosis of organism. Studies have found that miRNA is involved in the occurrence and development of many human diseases, including cancer^3^, cardiovascular disease^4^, diabetes^5^ and so on. However, its role in the process of liver fibrosis has not been well studied, and at present, the relationship between miRNAs and the development of liver fibrosis is gradually gaining attention.

Christoph et al.^6^ found that the serum level of miR-571 was significantly changed in patients with alcoholic or hepatitis C-induced cirrhosis, and the pro fibrogenic cytokines TGF-β and miR-571 were up-regulated in human hepatocytes and hepatic stellate cells. Yu et al. analyzed the gene expression profiles of 9 hepatocellular carcinoma samples and 9 normal liver tissues, and found a never reported miR-571 in hepatocellular carcinoma samples^7^. However, the effects and specific mechanisms of miR-571 on the development and progression of liver fibrosis require further study.

As an evolutionarily highly conserved signaling pathway, notch is widely involved in and finely regulates cell proliferation, differentiation and apoptosis processes^8^. With the development of molecular biology, the Notch pathway has been increasingly used in the study of several diseases, including tumors and fibrosis. In studies of fibrotic diseases in the human lung, kidney and liver, notch was found to act with TGF β1 to promote or degrade ECM, selectively mediating fibrogenesis^9–11^. It has also been shown that the levels of Notch3 and Jagged1 are positively correlated with the activation of HSCs in vivo in rats, and exposure of HSCs to Jagged1 stimulates α-SMA and collagen production to promote liver fibrogenesis^10^. In addition, the relationship between miR-571 and Notch signaling in liver fibrosis has also been proposed^12^. However, the relationship between miR-571 and Notch signaling pathway has not been studied, and the interaction in liver fibrosis has not been proposed. Therefore, to explore the correlation between miR-571 and Notch signaling pathway in the process of liver fibrosis is the focus of this study.

Based on previous research progress, this experiment mainly explores the expression of miR-571 in patients with liver fibrosis; explores the role of Notch3 in the occurrence and development of human liver fibrosis; finally explores the impact of miR-571 on the occurrence and development of liver fibrosis and the correlation with Notch3 signaling pathway.

## Research materials and experimental methods

### Patients

74 patients with hepatitis B, cirrhosis and concomitant liver fibrosis who were treated in our hospital from September 2018 to June 2019 were collected as study subjects. In addition, 19 cases of normal liver tissues outside of hepatic hemangioma were used as control group. Inclusion criteria: (1) Patients who met the diagnostic criteria for chronic hepatitis B from the 2015 edition of the guidelines for the prevention and treatment of chronic hepatitis B; (2) Patients aged ≥ 18 years and < 60 years; (3) Patients with complete data on clinical background.

Exclusion criteria: (1) Patients with hepatitis C and other viral hepatitis, autoimmune liver disease, alcoholic liver disease, liver cancer or other malignant tumors; (2) Patients with chronic infectious diseases (tuberculosis, hepatic hydatid, syphilis, human immunodeficiency virus infection); (3) Patients with comorbid diabetes, hypertension, coronary atherosclerotic heart disease, cerebrovascular disease; (4) Signed informed consent was not obtained from the patient; (5) Patients with incomplete clinical data.

This study was approved by the ethics committee of the College of Basic Medical Sciences, Guizhou Medical University, and written informed consent was obtained from all participants (Ethics No.: 2009035).

### HE staining

Liver tissue sections were subjected to HE staining. Routinely prepared paraffin sections were immersed in xylene for fixation for 30 min, dehydrated in 100%, 95%, 85%, 75% ethanol solution for 5 min each, rinsed in running water, stained by adding hematoxylin (DAKEWE, Beijing, China) for 10 min, immersed in 1% hydrochloric acid and rinsed with distilled water. Eosin staining (DAKEWE, Beijing, China) was added for 3 min, followed by dehydration in 75%, 85%, 95% and 100% ethanol solution for 2 min sequentially, xylene transparent treatment was added, neutral resin cover slips were added dropwise, placed and observed under a microscope, and five fields were selected to calculate the fibrosis area. Hepatocyte degeneration, necrosis, and inflammatory cell infiltration were observed, scored, and graded.

### Masson staining

Liver tissue sections were subjected to Masson staining. Routinely prepared paraffin sections were immersed in xylene for fixation for 30 min, dehydrated in 100%, 95%, 85%, 75% ethanol solution for 5 min each, then wash with distilled water for 2 min. Bouin solution (DAKEWE, Beijing, China) was fixed at 56 ℃ for 1 h, washed with flowing water until colorless, and then stained with azure blue for 5 min. Continue to use hematoxylin dyeing for 5 min, then use Fuchsin Acid Dye (DAKEWE, Beijing, China) for 10 min after washing with tap water. After washing with distilled water for 2 min, 1% phosphomolybdic acid was used to decolorize for 5 min; Further, brilliant green for 5 min and 1% glacial acetic acid for 5 min were used followed by dehydration in 75%, 85%, 95% and 100% ethanol solution for 2 min sequentially, xylene transparent treatment was added, neutral resin cover slips were added dropwise, placed and observed under a microscope, and five fields were selected to calculate the fibrosis area. Hepatocyte degeneration, necrosis, and inflammatory cell infiltration were observed, scored, and graded.

### Sirius red staining

Liver tissue sections were subjected to Sirius red staining. Routinely prepared paraffin sections were immersed in xylene for fixation for 30 min, dehydrated in 100%, 95%, 85%, 75% ethanol solution for 5 min each, then wash with distilled water for 2 min. It was treated with azure blue for 5 min, washed with distilled water for 2 min, dyed with Sirius red picric acid for 30 min, decolorized with 100% ethanol for 5 min, and sealed with neutral resin after xylene treatment. Leica DM 2500 polarizing microscope was used to observe the amount and degree of collagen deposition in the hepatic portal area, and the fibrosis stage was scored.

### Cell culture

Human hepatic stellate cells (LX-2) were purchased from American type culture collection (ATCC) and stored at − 80 °C. LX-2 cells were cultured in DMEM medium containing 10% FBS at 37 ℃, 5% CO_2_ incubator. After adherent growth, the cells were digested with 0.25% trypsin solution. When the cells grew to 80%, DMEM medium was added to stop digestion.

### Experimental grouping

Effects of Notch3 signaling on fibrosis in LX-2 cells, the following groups were set up: Notch3 upregulated group (rNotch3 group), Notch3 downregulated group (si-Notch3 group), control group, and scramble siRNA group. Recombinant Notch3 was used to induce Notch3 signaling and Notch3 short interference (si) RNA was used to downregulate Notch3 expression, respectively (Table 1).

**Table 1:** Different treatments of LX-2 cells.

Group	Notch3 signal processing	Group	MiR-571 processing
rNotch3 group	Recombinant Notch3 was used to induce Notch3 signaling	Mimics group	Transfected with miR-571 mimics
si-Notch3 group	Notch3 short interference (si) RNA was used to downregulate Notch3 expression	Inhibitor groups	Transfected with miR-571 inhibitor mimics
Control grouop	The cells are not treated at all	Control grouop	The cells are not treated at all
Scramble group	Meaningless sequence of Notch3	Negative control groups	Transfected with negative control mimics

To examine the effects of miR-571 on cell biology via the Notch signaling pathway, mimics groups (transfected with miR-571 mimics); negative control groups (transfected with negative control mimics); inhibitor groups (transfected with miR-571 inhibitor mimics); blank control groups were set up (Table 1).

### Transient transfection of cells

LX-2 cells in log phase were seeded into 6-well plates at approximately 1 × 10^5^–5 × 10^5^ cells per well in a 37 °C, 5% CO_2_ incubator for 24 h and used for transfection when the cells grew to 80% confluence. The transfection process was strictly carried out according to the biological transfection kit (ribo FECT™ CP transfection kit, Ruibo, Guangzhou, China). The transfection efficiency was observed by fluorescence microscope after 24 h.

### Apoptosis was assessed by Annexin V/PI staining

The cells in each group were cultured for 48 h, treated with 0.25% trypsin for 24 h, and then washed with phosphate buffer (PBS) for 3 times. Add 195 μL Annexin V-FITC (Invitrogen, MA, USA) binding solution to gently suspend the cells, then add 5 μL annexin V-FITC and mix well; finally add 10 μL PI staining solution to mix well. After mixing, the cells were placed at room temperature in dark for 15 min (the cells were resuspended for 3 times), and then placed in ice bath; the apoptosis rate was detected by flow cytometry.

### CCK-8 method was used to detect cell proliferation

The transfected cells (1 × 10^4^ cells/well) were seeded into 96 well plates, and at different time points, the cell number was determined using a cell counting CCK8 proliferation assay kit (Dojindo, Japan). 10 μL of CCK-8 solution was added to each well, and the absorbance at 450 nm was measured with a microplate reader. Inhibition rate = (1 − mean OD value of experimental group/mean OD value of control group) × 100%. Proliferation rate = (mean OD value at other time points/mean OD value at 0 h − 1) × 100%.

### Transwell assay for cell migration

The Transwell chamber was placed inside a 24 well plate, and the cell concentration was diluted into 5 × 10^5^/mL by adding 200 μL of cell suspension in the upper chamber and 500 μL of DMEM medium containing 10% FBS in the lower chamber, followed by incubation for 20 h at 37 °C, 5% CO_2_ cell culture incubator. Remove the upper chamber, add 600 μL of 4% paraformaldehyde, fix for 25 min at room temperature, then transfer the upper chamber to 1% crystal violet dye solution for 30 min at room temperature, PBS wash 2 times. Pictures were taken under a 100× microscope, and the number of cells in the field was recorded, and five different fields were averaged for each chamber, and all trials were repeated 3 times.

### Scratch healing assay

The logarithmic phase LX-2 cells were seeded in 24 well plates with the cell concentration of 2 × 10^5^ cells/mL. After 24 h of culture, a straight line was gently drawn at each empty position with a sterile gun head, washed twice with normal saline, and photographed for recording. After 24 h of culture, the scratch and coincidence were observed under the microscope.

### Real time fluorescence quantification PCR (RT-PCR)

Total cellular RNA was adequately extracted using TRIzol kit (Invitrogen, MA, USA), and then the RNA was reverse transcribed to cDNA using the reverse transcription kit for RT-PCR reaction using cDNA as template. PCR reaction conditions were 95 °C for 5 min; 95 °C for 15 s, 60 °C for 32 s, a total of 40 cycles were performed. Gene relative quantification was performed using the 2^−ΔΔCT^ method with GAPDH as an internal reference gene on the CFX Manager 3.0 software (Bio-Rad, CA, USA). RT-PCR primer sequences are shown in Table 2.

**Table 2:** RT-PCR primer sequences.

Gene names	Primer sequences (5′–3′)
miR-571 forward primer	ACACTCCAGCTGGGAGAGTTGGCCATCTG
miR-571 reverse primer	TGGTGGTCGTGGAGTCG
TGF-β1 forward primer	GTGGACCGCAACAACGCAATCTATG
TGF-β1 reverse primer	GGCACTGCTTCCCGAATGTCTGA
SAMD3 forward primer	CAGGGCTTTGAGGCTGTCTA
SAMD3 reverse primer	CTGGCATCTTCTGTGGTTTC
α-SMA forward primer	TGGTATTGTGCTGGACTCTG
α-SMA reverse primer	CCATCAGGCAGTTCGTAG
Collagen I forward primer	CAGCCGCTTCACCTACAGC
Collagen I reverse primer	TTGTATTCAATCACTCTCCTTGCC
Notch3 forward primer	AGGCTACCTTGGCTCTGCTGAA
Notch3 reverse primer	CAGCCTGTCCAAGTGATCTGTGA
Jagged1 forward primer	GATTTCCTGGTTCCTCTGCTG
Jagged1 reverse primer	CATTGTTGGTGGTGTTGTCCT
GAPDH forward primer	TGTGTCCGTCGTGGATCTGA
GAPDH reverse primer	TTGCTGTTGAAGTCGCAGGAG

### Western blot analysis

Total proteins from each group were extracted with RIPA lysis buffer (Biyuntian, Beijing, China) mammalian protein extraction reagent. The total protein concentration was determined using a BCA protein assay kit (Kaiji, Nanjing, China). After separation of proteins by SDS-PAGE, the membranes were transferred to PVDF membranes (Millipore, MA, USA) and blocked with 5% nonfat dry milk solution for 2 h at room temperature, and the primary antibodies of TGF-β1, SMAD3, α-SMA, Collagen I, Notch3, Jagged I were diluted at a concentration (Rabbit anti tubulin antibody, 1:1000) overnight at 4 °C, the primary antibodies were used; the membranes were washed with TBST for 8 min and 3 times. After washing the membrane, HRP labeled dilutions of the corresponding secondary antibodies (Goat anti rabbit IgG, 1:5000) were added and incubated at 37 °C for 50 min ∼ 3 h; the membrane was washed three times for 8 min each using TBST; the ELC luminescent solution (Aillipore, MA, USA) was used for color exposure, and GAPDH was used as an internal reference to analyze the relative protein expression.

### Statistical analysis

SPSS 21.0 (IBM, Armonk, New York, USA) and origin 9.1 (Microcal, Northampton, Massachusetts, USA) statistical software were used for data analysis. The measurement data were expressed as mean ± standard deviation ($\overline{\chi } \pm {\text{s}}$). Student's t test was used for statistical analysis. One way ANOVA was used for comparison. *P* < 0.05 was considered as statistically significant.

### Statement

All methods were carried out in accordance with relevant guidelines and regulations.

## Results

### Correlation between liver fibrosis and miR-571, Notch3 and Jagged1 expression

#### Liver fibrosis status of the patients

**Figure 1:**
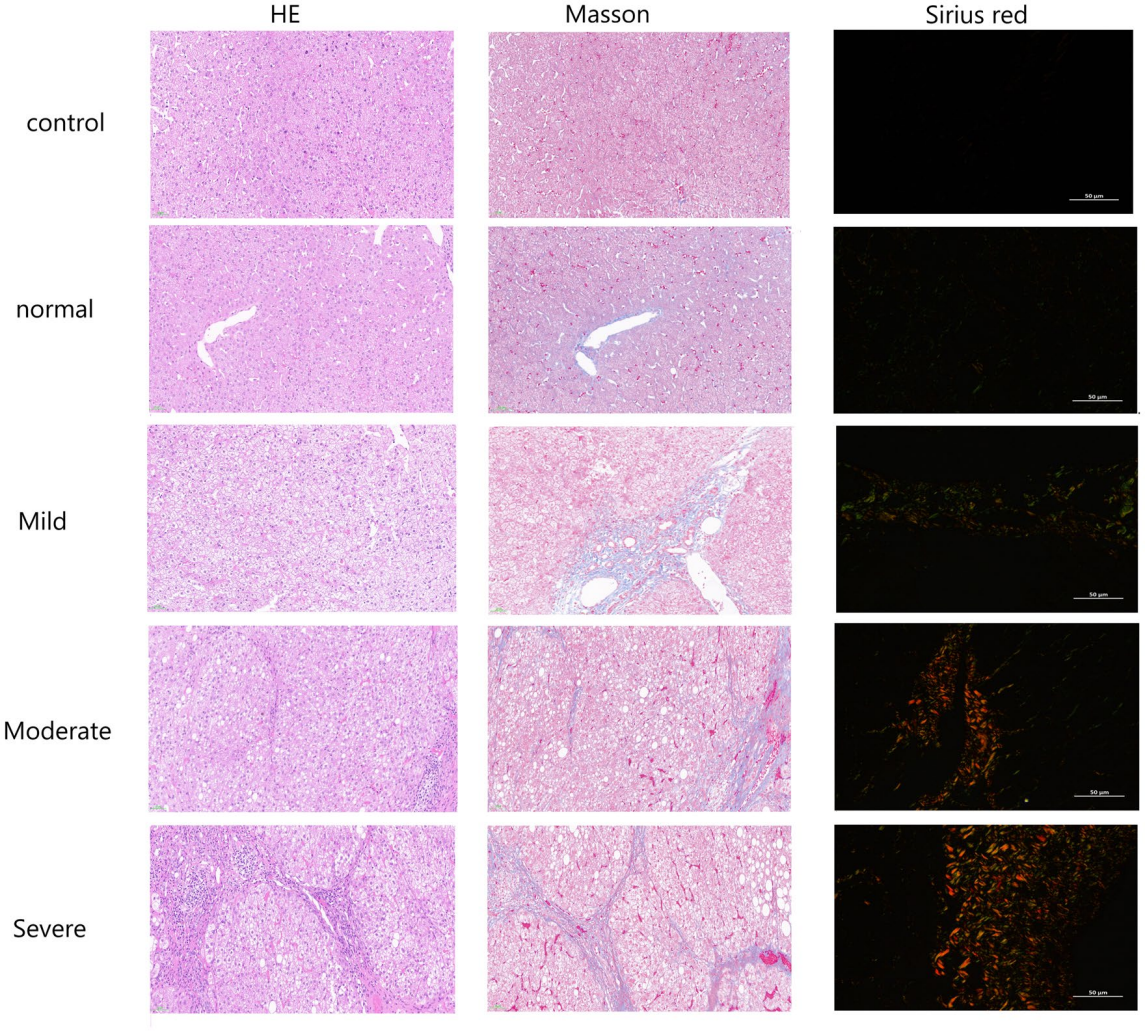
Pathological staining results of different degrees of liver fibrosis and normal liver tissue. There were three different staining results, HE staining, Masson staining and Sirius red staining. The control group was the normal liver tissue outside the hepatic hemangioma. Normal group was liver tissue with chronic hepatitis but without liver fibrosis. Mild group was chronic liver disease with mild liver fibrosis. The moderate group was chronic liver disease with moderate liver fibrosis. The severe group was the liver tissue with chronic liver disease and severe hepatic fibrosis.

Masson and he staining results showed that the structure of hepatic lobules in normal liver tissue and non fibrosis liver tissue was complete, and the arrangement of hepatic cords was regular. The mild group showed swelling of hepatocytes and a small amount of focal necrosis. In the moderate group, the liver tissue showed structural disorder of hepatic lobules, obvious round vacuoles in liver cells, steatosis, aggravation of necrosis, infiltration of inflammatory cells and proliferation of fibrous tissue. In severe group, a large number of fibrous bands and some fibrous septa were formed. The results of Sirius red showed that the collagen fibers in the normal group were limited to the vascular wall of the portal area, and the red type I collagen fibers, a small amount of type II collagen fibers and green type III collagen fibers could be observed under the polarizing microscope. With the aggravation of liver fibrosis, collagen deposition was observed under polarizing microscope, including a large number of red type I collagen fibers, green type III collagen fibers, a small number of type II collagen fibers and light yellow type IV collagen fibers. There were 11 normal liver tissues in this experiment, 17 patients with mild liver fibrosis, 26 patients with moderate liver fibrosis, and 20 patients with severe liver fibrosis; the HE staining pictures of liver fibrosis were shown in Fig. 1.

### Differential expression of miR-571 in patients with different degrees of liver fibrosis

**Figure 2:**
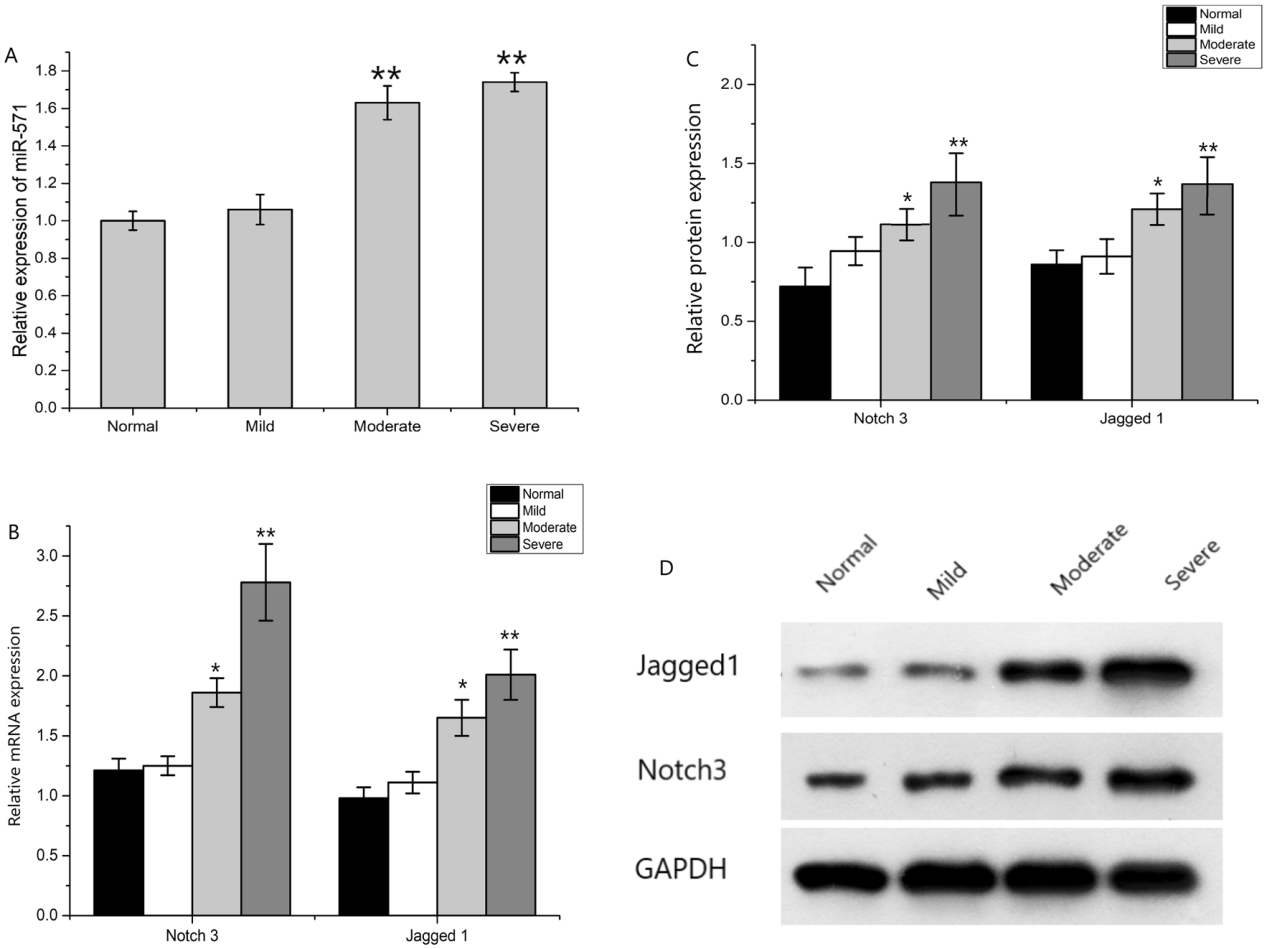
Expression differences of miR-571, Notch3 and Jagged1 in different liver fibrosis tissues (**A**) Differential tissue miR-571 expression in patients with liver fibrosis at different stages of progression. miR-571 is associated with the degree of liver fibrosis, and the more severe the fibrosis, the higher the miR-571 expression. *P < 0.05, **P < 0.01. (**B**) Difference of mRNA expression of Notch3 and Jagged1 in liver fibrosis tissues of different degrees. (**C**, **D**) Difference of protein expression of Notch3 and Jagged1 in liver fibrosis tissues of different degrees. With the aggravation of liver fibrosis, the expression of Notch3 and Jagged1 increased gradually. *P < 0.05, **P < 0.01.

The expression of miR-571 increased in patients with liver fibrosis, and was correlated with the severity of liver fibrosis. The higher the degree of liver fibrosis, the higher the expression of miR-571; there was significant difference between patients with severe and moderate liver fibrosis and normal and mild patients (*P* < 0.05), but there was no significant difference between mild patients and normal (*P* > 0.05) (Fig. 2A).

### Differential expression of Notch3 and Jagged1 in patients with different degrees of liver fibrosis

RT-PCR and Western blot were used to detect the expression of Notch3 and Jagged1 in different degrees of liver fibrosis. The results showed that there was no significant difference in Notch3 and Jagged1 between mild patients and patients without liver fibrosis (*P* > 0.05), but the expression of Notch3 and Jagged1 in moderate and severe patients was significantly higher than that in patients without liver fibrosis (*P* < 0.05) (Fig. 2B–D).

### Effect of Notch3 signaling pathway on the expression of fibrogenic factor

#### Effects of Notch3 signaling pathway on the expression of fibroblast mRNA

**Figure 3:**
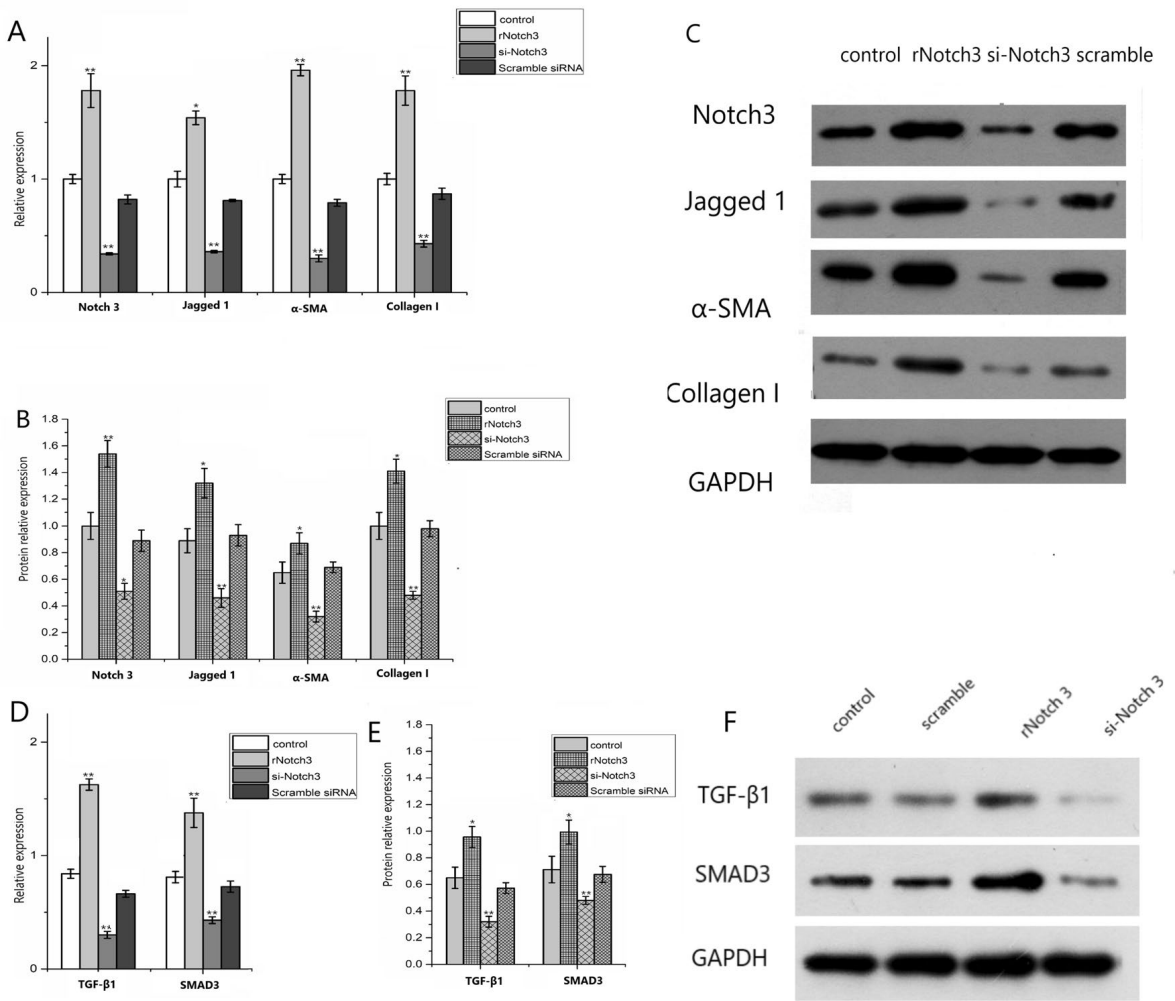
Effect of Notch3 signaling on expression of profibrogenic factors. (**A**) Differences in mRNA expression of Notch3 and profibrogenic factors; (**B**, **C**) Differential expression of Notch3 and profibrogenic factor; (**D**) Effect of Notch3 on TGF-β1 and Smad3 mRNA expression. (**E**, **F**) Effect of Notch3 on TGF-β1 and Smad3 protein expression. Recombinant Notch3 was used to induce Notch3 signaling and Notch3 short interference (si) RNA was used to downregulate Notch3 expression, respectively. The mRNA and protein expressions of the receptor Jagged1 and the pro-fibrinogen were up-regulated after Notch3 up-regulation, and the mRNA and protein expressions of the receptor Jagged1 and the pro-fibrinogen were down-regulated after Notch3 down-regulation. *P < 0.05, **P < 0.01.

The mRNA expression of Notch3 and Jagged1 was significantly increased in the rNotch3 group compared with the control group, and the expression of Notch3 and Jagged1 was significantly decreased in the si-Notch3 group (*P* < 0.05), indicating that the upregulation of Notch3 signaling and interference are effective. The mRNA expression of α-SMA and collagen I was significantly increased in the rNotch3 group compared with the control group and scramble siRNA group, and decreased in the si-Notch3 group (*P* < 0.05) (Fig. 3A). Similarly the cytokine TGF-β1 and Smad3 showed the same trend in mRNA expression (Fig. 3D). The mRNA expressions of TGF-β1 and Smad3 in rNotch3 group were significantly up-regulated, while the expression of si-Notch3 group was significantly down-regulated (*P* < 0.05).

### Effect of Notch3 signaling on profibrogenic protein expression

Compared with the control group and scramble siRNA group, the protein expressions of α-SMA and collagen I in the Notch3 up regulation group (rnotch3 group) were significantly increased (*P* < 0.05), while the protein expressions of α-SMA and collagen I in the Notch3 down regulation group (siRNA group) were significantly decreased (*P* < 0.05). The effects of Notch3 signaling pathway on the expression of fibrogenic factors are shown in Fig. 3B,C. TGF-β1 and Smad3 protein expressions were significantly up-regulated in rNotch3 group, and down-regulated in si-Notch3 group (*P* < 0.05) (Fig. 3E,F).

### Effect of miR-571 on biological behavior of human hepatic stellate cells through Notch3 signaling pathway

#### Effect of miR-571 on cell proliferation

**Figure 4:**
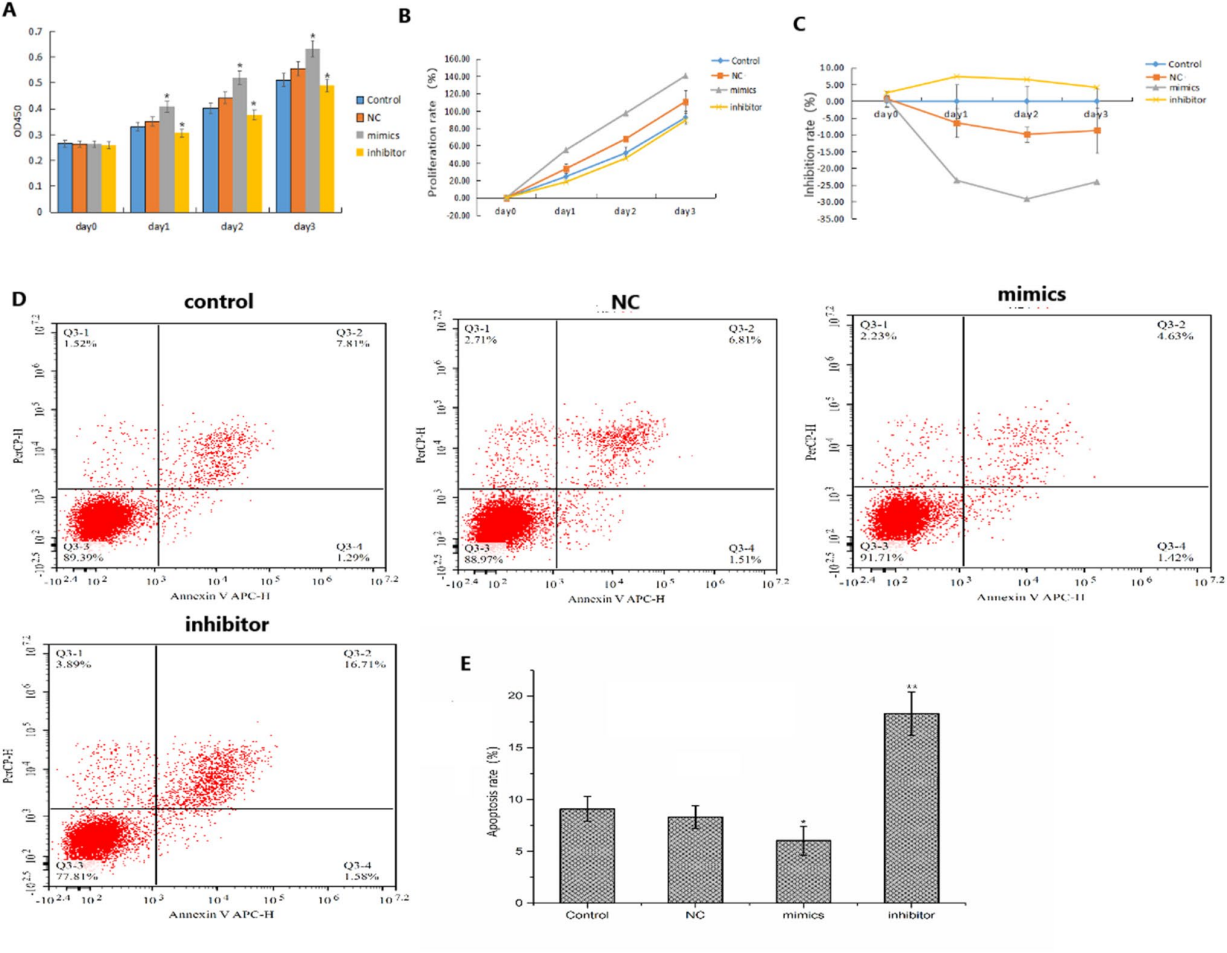
Effect of miR-571 on proliferation and apoptosis of human hepatic stellate cells. (**A**–**C**) Effect of miR-571 on proliferation of human hepatic stellate cells. Experimental groups (transfected with miR-571 mimics); negative control groups (transfected with negative control mimics); inhibitor groups (transfected with miR-571 inhibitor mimics); blank control groups were set up. The up-regulation of miR-571 promoted the proliferation of human hepatic stellate cells. Downregulation of miR-571 can inhibit the proliferation of human hepatic stellate cells. (**D**, **E**) Effect of miR-571 on apoptosis of human hepatic stellate cells. The lower-left quadrant of each image represents normal cells, and PI and AV staining were negative (AV−/pI−). The lower right quadrant represents apoptotic cells. AV positive and PI negative (AV+/PI−). The upper right quadrant shows necrotic cells and positive for AV and PI staining (AV+/PI+). Up regulation of miR-571 inhibits apoptosis of human hepatic stellate cells. *P < 0.05, **P < 0.01.

The proliferation and inhibition rates of cells were different in different groups on 1d, 2d and 3d; Proliferation was significantly higher in the miR-571 mimics group than in the control and NC groups, and significantly lower in the miR-571 inhibitors group than in the control and NC groups (*P* < 0.05); however, with respect to inhibition rates, the miR-571 mimics group had a significantly lower proliferation rate than the control group and the inhibitors group had a significantly higher proliferation rate (*P* < 0.05) (Fig. 4A–C).

### Effect of miR-571 on human hepatic stellate cell apoptosis

The apoptosis rate of miR-571 inhibitors group was 18.28 ± 1.21%, which was significantly higher than that of the control group (*P* < 0.05), and it was mainly late apoptosis; the apoptosis rate of miR-571 mimics group was 6.05 ± 0.9%, which was significantly lower than that of the control group (*P* < 0.05) (Fig. 4D,E).

### Effect of miR-571 on migration of human stem stellate cells

**Figure 5:**
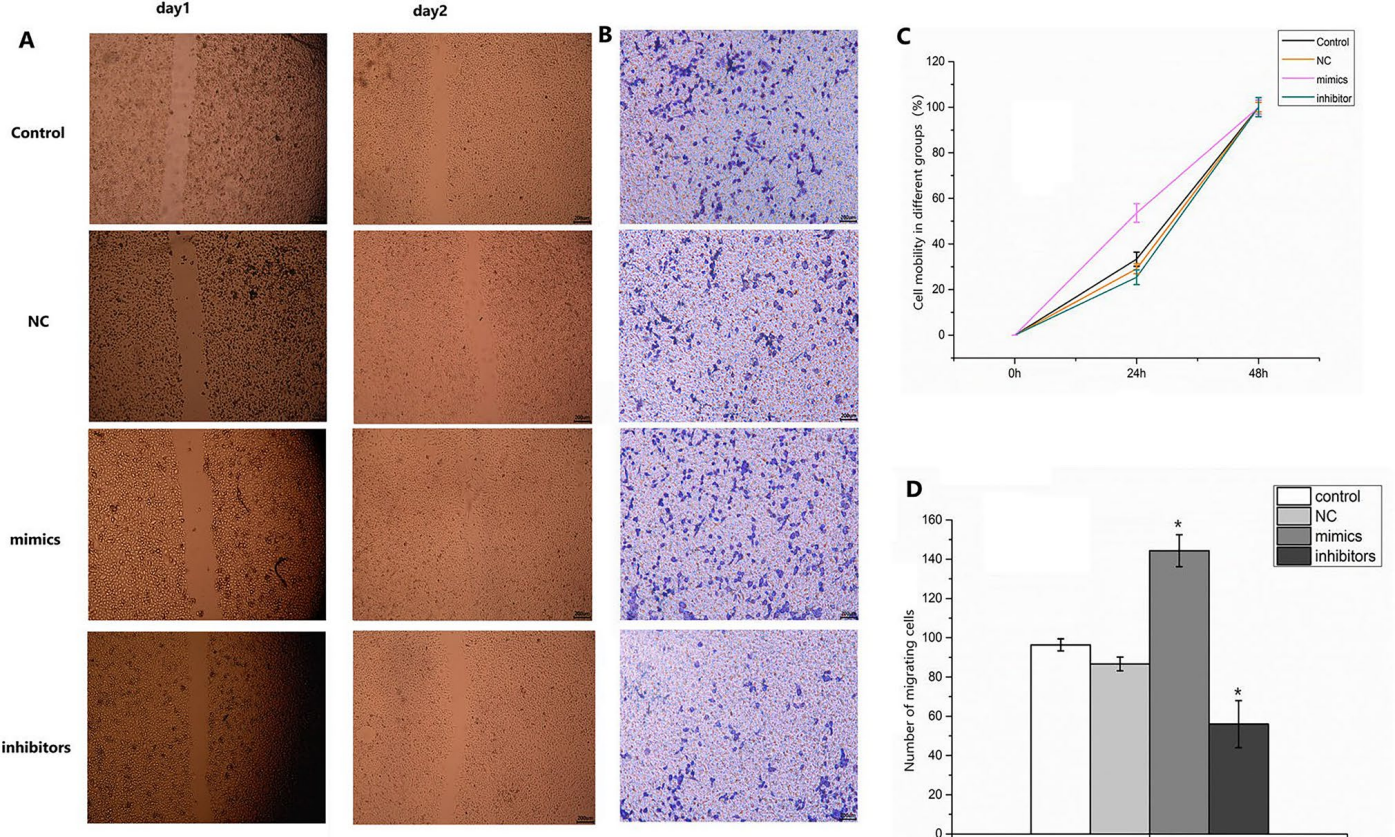
Effect of miR-571 on migration of human hepatic stellate cells. (**A**, **C**) The migration rate of cells in different groups was detected by scratch test; (**B**, **D**) Transwell assay was used to detect cell mobility. Experimental groups (transfected with miR-571 mimics); negative control groups (transfected with negative control mimics); inhibitor groups (transfected with miR-571 inhibitor mimics); blank control groups were set up. Up regulation of miR-571 promotes migration of human hepatic stellate cells. *P < 0.05, **P < 0.01.

The effect of miR-571 on human hepatic stellate cell migration was examined by cell scratch assay and Transwell assay (Fig. 5). A significant increase in cell migration was observed in the miR-571 mimics group compared with the control and NC groups, and a significant decrease in cell migration was observed in the miR-571 inhibitors (*P* < 0.05); this result suggests that miR-571 is able to promote human hepatic stellate cell migration.

### Effects of miR-571 on Notch3 signaling pathway and expression of fibroblasts

**Figure 6:**
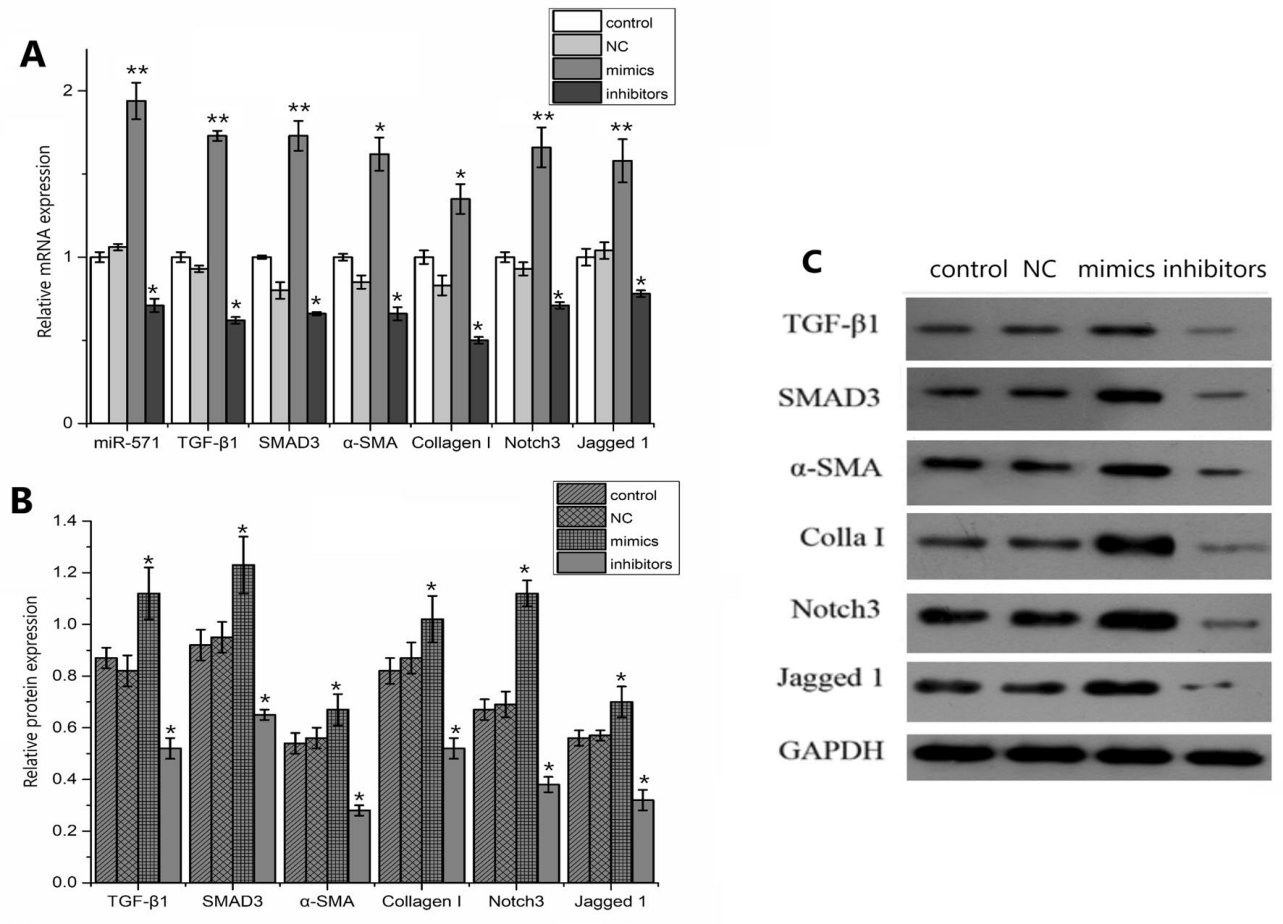
Effects of miR-571 on Notch3 signaling pathway and expression of fibrogenic factors. (**A**) Effects of miR-571 on Notch3 signaling pathway and expression of fibroblast mRNA; (**B**, **C**) effects of miR-571 on Notch3 signaling pathway and the expression of fibroblast protein. Experimental groups (transfected with miR-571 mimics); negative control groups (transfected with negative control mimics); inhibitor groups (transfected with miR-571 inhibitor mimics); blank control groups were set up. Up regulation of miR-571 promotes the expression of Notch3 signaling pathway and fibrogenic factor mRNA and protein. *P < 0.05, **P < 0.01.

The expression of miR-571 in the miR-571 mimics group was significantly increased, while that in the inhibitor group was significantly decreased, indicating that the up-regulation and down-regulation of miR-571 were effective. Compared with the control group and NC group, the mRNA and protein expressions of Notch3 and Jagged1 in miR-571 mimics group were significantly increased (*P* < 0.05), which indicated that miR-571 had a certain regulatory effect on Notch3 signaling pathway. Compared with the control group and NC group, the expressions of TGF-β 1, fibrogenic factors Smad3, α-SMA and collagen I were significantly increased in miR-571 mimics group, while the expressions of TGF-β1, fibrogenic factors Smad3, α-SMA and collagen I were significantly decreased in inhibitors group (*P* < 0.05) (Fig. 6).

## Discussion

Liver fibrosis is the destruction of liver parenchymal cells and self repair of tissue caused by many factors, which is characterized by excessive production and deposition of extracellular matrix mainly composed of collagen. It is the only way for various chronic liver diseases to develop into cirrhosis. When chronic liver injury occurs, resting hepatic stellate cells (HSC) become active, which is the central link of liver fibrosis^2^. The occurrence and development of liver fibrosis is a multifactorial and multifaceted process, resulting from the imbalance of several related genes.

MiRNAs have been found to be involved in the development of many human diseases, and their roles in the process of liver fibrosis have gradually become a research focus^13^. Guo et al.^14^ found that 13 miRNAs were up-regulated and 22 down regulated during the activation of HSCs. Jensen et al.^15^ further demonstrated that miR-199a-5p, miR-182, miR-183, and miR-200a-5p were significantly upregulated in F3 and F4 stages of liver fibrosis compared with the early stages F1 and F2, and miR-148-5p, miR-1260b, miR-122-3p, and miR-378i were most significantly downregulated from early to advanced stages of liver fibrosis. This suggests that by studying the differential expression of miRNAs during liver fibrosis, it will be helpful for understanding the mechanisms of the development and progression of liver fibrosis, as well as for finding novel molecular regulatory targets of liver fibrosis.

A large number of experiments have confirmed that miRNA is closely related to the occurrence and development of liver fibrosis^16–18^. Some studies have reported that miR-181b can promote HSC activation by mediating the PTEN/Akt pathway to affect the initiation and progression of liver fibrosis^19^. In addition, miR-200s, which can directly bind p85 α and inhibit the activation of the PI3K/Akt pathway through Fog2, can lead to HSC growth and migration, serving as a potential marker for HSC activation and liver fibrosis progression^20^. Up regulation of miR-9a-5p can induce HSC proliferation, migration and activation^21^. All the above studies indicate that miRNA is closely related to the occurrence and development of liver fibrosis, and the regulation of HSC activation, proliferation, apoptosis and migration by miRNA is one of the mechanisms of the occurrence and development of liver fibrosis, which may be mediated by various signaling pathways.

The relationship between Notch3 signaling pathway and liver fibrosis has also been deeply studied. Zheng et al. found that Notch3 may be involved in liver fibrosis by regulating the activation of hepatic stellate cells (HSCs); after downregulation of Notch3 by lentivirus transfected cells, Notch3, Jagged1, Hes1 and α-SMA were downregulated, and in mice with in vivo inhibition of Notch3, this downregulation was accompanied by improved liver fibrosis^22^. This result was also confirmed by Chen et al.^23^. In this experiment, we found that the expression of fibroblast α-Sam and collagen I increased significantly after up regulating Notch3 signal in human LX-2 cells, but down regulating Notch3 signal had the opposite effect. The results also confirmed that Notch3 had the same effect in human hepatic stellate cells.

Christoph et al.^24^ Systematically analyzed the miRNA serum levels of cirrhotic patients from different entities and compared them with the expression profiles of healthy controls. The results showed that the expression of miR-571 was up-regulated. In addition, there are few studies on miR-571. We further explore the role of miR-571 in the process of liver fibrosis. First of all, we studied the species with miR-571. The results showed that as long as *human*, *Pan troglodytes*, *Gorilla gorilla*, *Pongo pygmaeus* and *Macaca mulatta* found homologous sequences, no homologous sequences were found in mice, rats and rabbits; so human hepatic stellate cells were chosen for this study. In a study of the differences in miR-571 expression between liver tissues from patients with and without liver fibrosis, miR-571 was found to be upregulated in patients with liver fibrosis and to be associated with different stages of fibrosis. This result suggests that miR-571 plays a role in the progression of liver fibrosis, so we further explored the regulatory mechanism.

The development and progression of liver fibrosis is closely associated with human hepatic stellate cell activation based on the results, we first explored the effects of miR-571 on human hepatic stellate cell proliferation, apoptosis and migration. Found that miR-571 was able to promote cell proliferation and migration, as well as inhibit apoptosis; this suggests that miR-571 is able to promote the progression of liver fibrosis. In addition, miR-571 can regulate the expression of fibrogenic factors to regulate the occurrence and development of liver fibrosis. miR-571 may be associated with the Notch signaling pathway, and it was found to down regulate the expression of miR-571 and the expression of Notch3 and its receptor Jagged1, which suggests that miR-571 may mediate Notch3 signaling to regulate the activation of human stem stellate cells^12^.

## Conclusion

MiR-571 can promote the activation of human hepatic stellate cells and the expression of fibrogenic factors through Notch3 signaling pathway. Because miR-571 is homologous in mice, no animal experiment was conducted in this experiment; in addition, the progress of liver fibrosis involves multiple genes and cellular signaling pathways, and the regulatory role of miR-571 may also involve multiple signaling networks, which needs further experimental exploration in the future.

## Supplementary Information

Supplementary Information.
