# Author Correction: MiR-571 affects the development and progression of liver fibrosis by regulating the Notch3 pathway

**DOI:** 10.1038/s41598-025-00191-3

**Published:** 2025-05-12

**Authors:** Shuo Cong, Yongmei Liu, Yi Li, Yu Chen, Rui Chen, Baofang Zhang, Lei Yu, Yaxin Hu, Xueke Zhao, Mao Mu, Mingliang Cheng, Zhi Huang

**Affiliations:** 1 School of Basic Medicine Sciences, Guizhou Medical University, 9 Beijing Road, Guiyang, Guizhou China; 2 Clinical Laboratory Center, Guizhou Cancer Hospital, 1, Beijing West Road, Guiyang, Guizhou China; 3 Clinical Laboratory Center, The Affiliated Hospital of Guizhou Medical University, 28, Guiyi Street, Guiyang, Guizhou China; 4 College of Medical Laboratory, Guizhou Medical University, 28, Guiyi Street, Guiyang, Guizhou China; 5 Department of Acupuncture and Moxibustion, The Affiliated Hospital of Guizhou Medical University, 28, Guiyi Street, Guiyang, Guizhou China; 6 Department of Infectious Diseases, The Affiliated Hospital of Guizhou Medical University, 28, Guiyi Street, Guiyang, Guizhou China; 7 Department of Obstetrics and Gynecology, Maternal and Child Health Hospital of Guiyang Province, 63 Ruijin South Road, Yunyan District, Guiyang City, Guizhou Province China; 8 Prenatal Diagnosis Center, The Affiliated Hospital of Guizhou Medical University, 9 Beijing Road, Guiyang City, Guizhou China; 9 Department of Interventional Radiology, The Affiliated Baiyun Hospital of Guizhou Medical University, Guiyang, 550005 P. R. China

Correction to: *Scientific Reports* 10.1038/s41598-021-00638-3, published online 08 November 2021

The original version of this Article contained an error in Figure 5B, where the image for ‘NC’ group was a duplication of the ‘Control’ group image. The original Figure 5 and accompanying legend appear below.

**Fig. 5:**
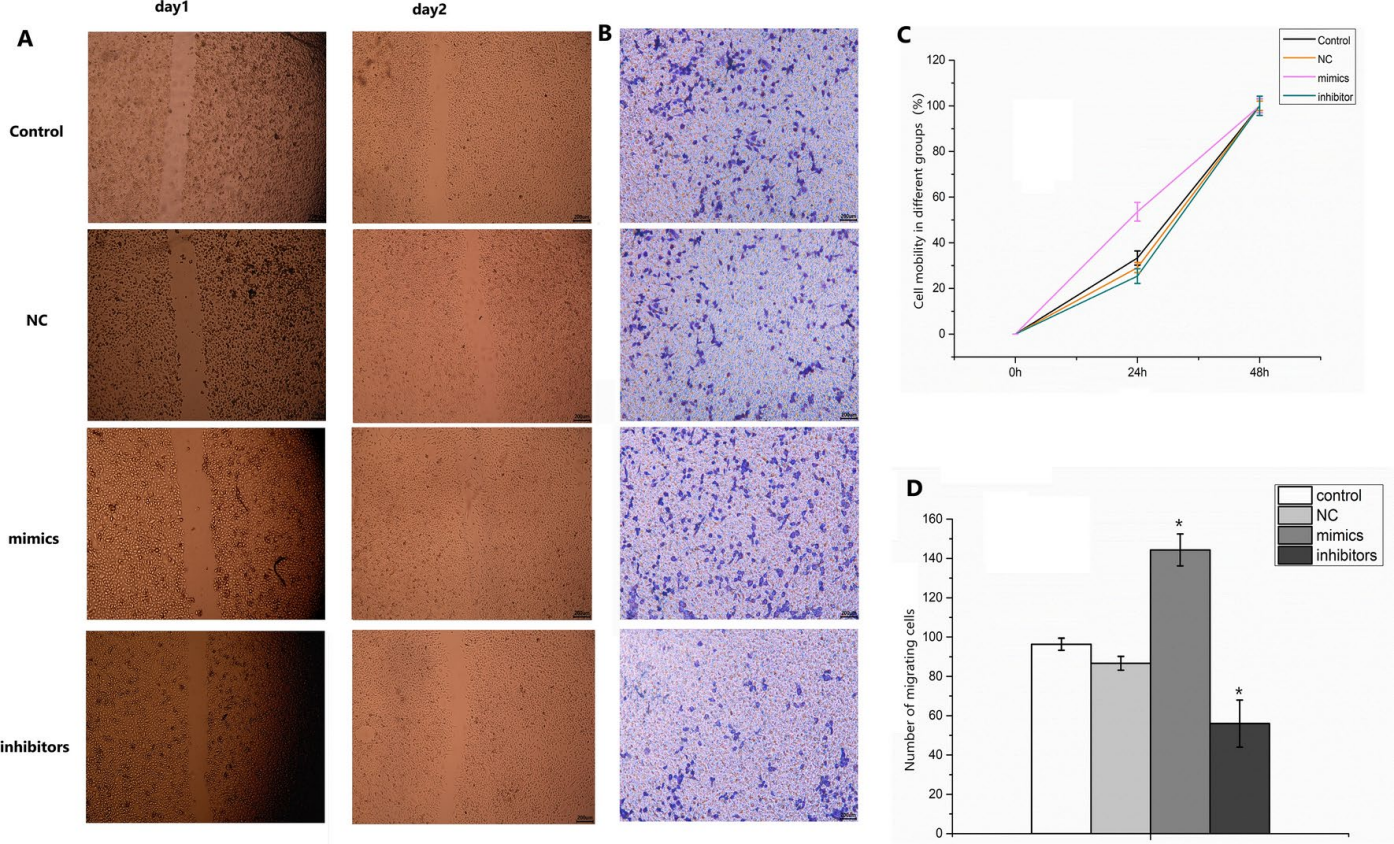
Effect of miR-571 on migration of human hepatic stellate cells. (**A**, **C**) The migration rate of cells in different groups was detected by scratch test; (**B**, **D**) Transwell assay was used to detect cell mobility. Experimental groups (transfected with miR-571 mimics); negative control groups (transfected with negative control mimics); inhibitor groups (transfected with miR-571 inhibitor mimics); blank control groups were set up. Up regulation of miR-571 promotes migration of human hepatic stellate cells. *P < 0.05, **P < 0.01.

The original Article has been corrected.

