# Supplementary material for: MiR-571 affects the development and progression of liver fibrosis by regulating the Notch3 pathway
Source: Sci Rep. 2021 Nov 8;11:21854. doi: 10.1038/s41598-021-00638-3 (PMC8575893; doi:10.1038/s41598-021-00638-3)
Supplement: Supplementary file 1 — Supplementary Information. [file 41598_2021_638_MOESM1_ESM.docx]

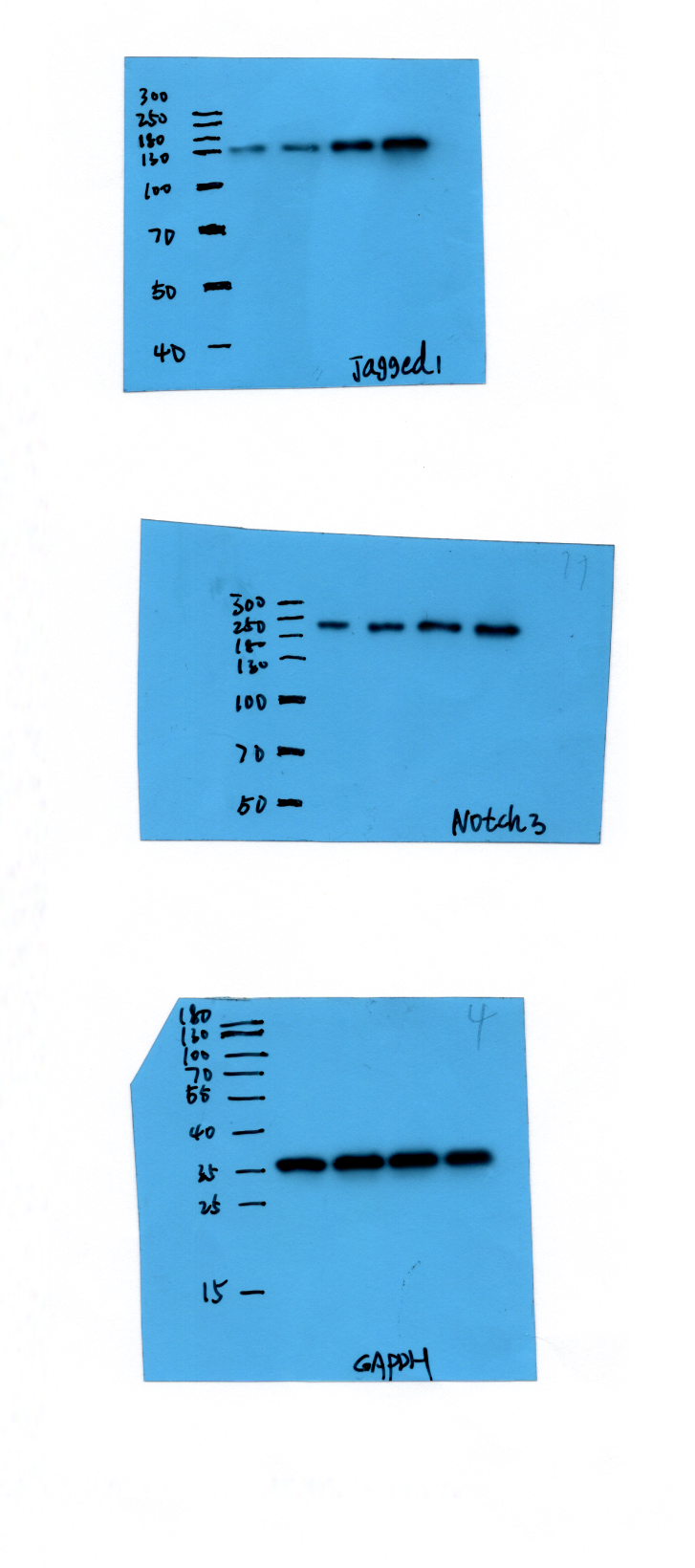


Supplementary Figure 1 Figure 2 original glue diagram. The protein expression of Jagged1 and Notch3 was detected by Western bolt. The expressionof Jagged1 and Notch3 increased with the deepening of liver fibrosis


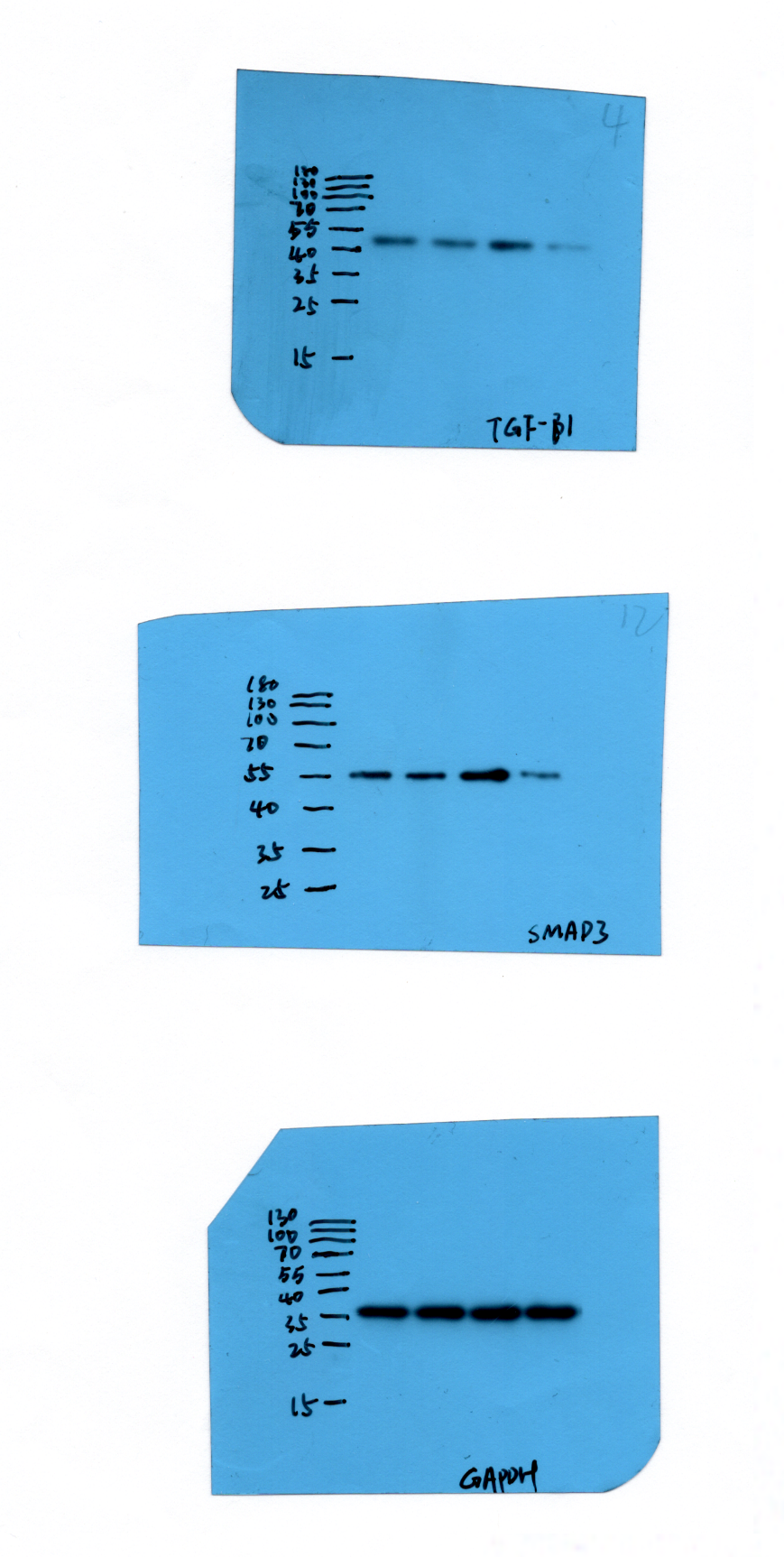


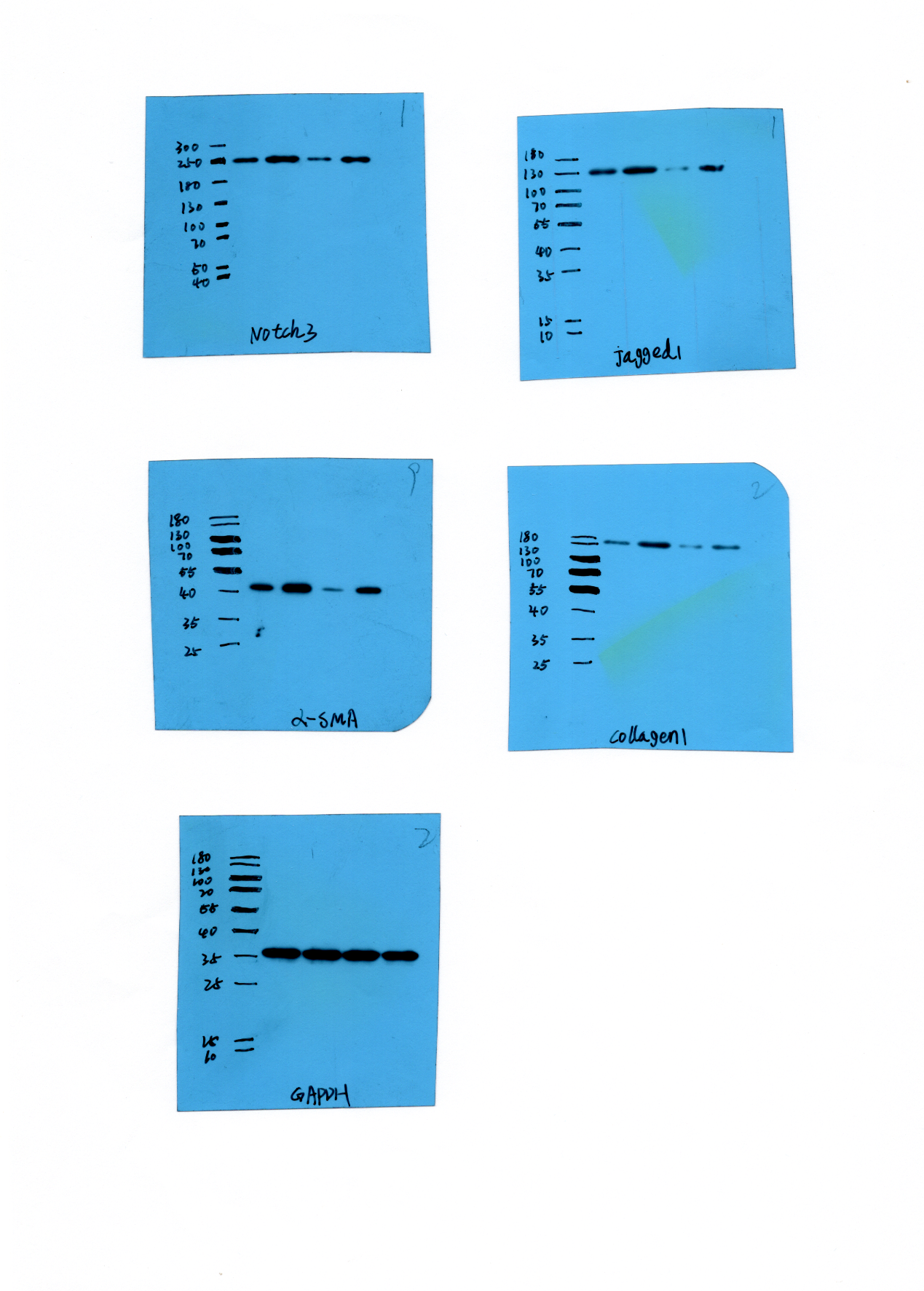


Supplementary Figure 2 Figure 3 original glue diagram. The expression of and collagen I, Notch3, Jagged1 α- SMA, TGF-β1 and SMAD3 in Notch3 up-regulated group, down-regulated group and control group.


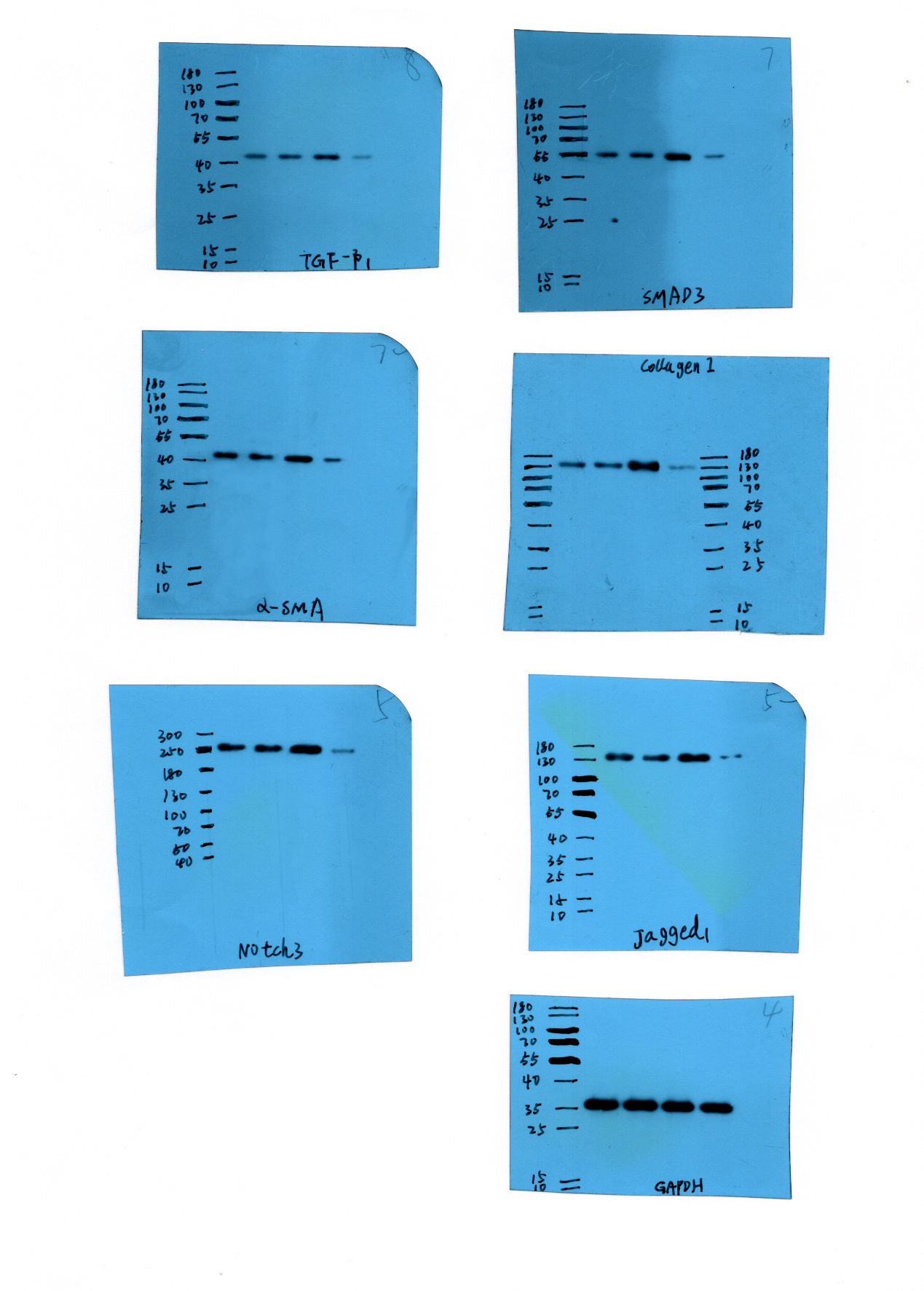


Supplementary Figure 3 Figure 6 original glue diagram. Expression of related proteins in miR-571 overexpression group, interference group and control group.
